# Hospital-based health technology assessment of central dialysis fluid delivery system for hemodialysis patients

**DOI:** 10.1186/s12882-025-04484-7

**Published:** 2025-10-27

**Authors:** Wendi Cheng, Haiyin Wang, Jiangzi Yuan, Ying Li, Yashuang Luo, Yuyan Fu, Leyi Gu, Jun Xue, Chunlin Jin, Yin Zheng

**Affiliations:** 1 Shanghai Health Development Research Center, (Shanghai Medical Information Center), Shanghai, China; 2 Department of Nephrology, Huashan Hospital, Fudan University, Shanghai, China; 3 Department of Nephrology, Baoshan Branch of Renji Hospital, Shanghai Jiao Tong University School of Medicine, Shanghai, China; 4 Department of Nephrology, Shanghai Jiading District Central Hospital, Shanghai, China; 5 Department of Nephrolgoy, Renji Hospital, Shanghai Jiao Tong University School of Medicine, Shanghai, China

**Keywords:** Central dialysis fluid delivery system (CDDS), Single-patient dialysis fluid delivery system (SPDDS), Hemodialysis, Hospital-based health technology assessment (HB-HTA), Economic value, Micro-costing

## Abstract

**Background:**

This study employed a hospital-based health technology assessment (HB-HTA) to evaluate the economic value of the central dialysis fluid delivery system (CDDS) compared to the single-patient dialysis fluid delivery system (SPDDS) in hemodialysis. The findings offer evidence to support hospital procurement and clinical implementation decisions regarding CDDS.

**Methods:**

A comparative cost analysis was conducted using micro-costing data collected from three hospitals. Costs were estimated for both CDDS and SPDDS, and economic efficiency was evaluated across multiple hemodialysis machine configuration scales. The analysis included total costs, cost structures, and cost savings, along with their constituent components, to identify scenarios in which CDDS provides economic advantages.

**Results:**

In a dialysis center with 6 hemodialysis machines operating two shifts per day and serving 12 patients daily, CDDS reduced costs by 0.05% compared to SPDDS. As the number of machines increased from 6 to 50, the cost reduction rate rose from 0.05% to 21.08%, demonstrating enhanced economic benefits at larger scales. In a 50-machine center, CDDS saved approximately RMB 84 per treatment session and RMB 2,601,108 annually. The majority of cost savings came from reductions in consumables (74%) and labor (24%). One-way sensitivity analysis confirmed that CDDS consistently yielded cost savings, supporting the robustness of the costing model.

**Conclusion:**

CDDS demonstrates significant economic value over SPDDS by reducing consumable and labor costs, proving to be a cost-effective solution in dialysis centers with 6 or more machines serving at least 12 patients daily. Clinical engineers play a key role in its implementation. CDDS is suitable for promotion in medium- to large-scale dialysis centers in China.

**Supplementary Information:**

The online version contains supplementary material available at 10.1186/s12882-025-04484-7.

## Background

Hospital-based health technology assessment (HB-HTA) refers to the evaluation of various health technologies specifically tailored to a hospital’s environment to support management decisions. HB-HTA provides hospital administrators with key evidence and analyses to assess the need for introducing new technologies, avoid the adoption of unsuitable technologies, and reduce the use of unnecessary ones, thereby enhancing the efficiency of hospital health resource allocation [1]. Compared to developing countries, HB-HTA receives more attention in developed nations, particularly in Europe and North America [2–11]. In recent years, interest in HB-HTA has increased in China, and related projects have provided scientific decision-making support for hospital adoption and management of new technologies. However, the number of researchers and institutions in this field remains limited, and the scope of research is narrow. In the future, more investment and encouragement for hospitals to conduct HB-HTA will be needed, along with the exploration of a system suitable for China’s national conditions to enhance the scientific nature of hospital decision-making [12].

In March 2018, the Medical Administration Center under the National Health Commission initiated the first phase of HB-HTA pilot projects in seven hospitals, followed by a second phase in March 2019 involving 23 hospitals [13], thereby creating a favorable policy environment for HB-HTA in China.

In recent years, end-stage renal disease (ESRD) has emerged as a major global health challenge due to its high prevalence and mortality rates [14, 15]. Hemodialysis (HD) remains the primary blood purification therapy for ESRD patients. China has the largest number of maintenance hemodialysis patients worldwide. At the 2024 Academic Annual Meeting of the Chinese Nephrology Association (CNA), academician Xiangmei Chen presented data from the China Research Data Services Platform. As of December 2023, there were 7,512 hemodialysis centers in China, with 916,600 patients, representing a prevalence rate of 635 per million population. Against this backdrop, China is urgently needed to reduce dialysis costs, improve efficiency, ensure high-quality dialysis, and lower overall healthcare expenditures.

The quality of dialysate and the efficiency of dialysate delivery systems are critical for ensuring cost-effective and high-quality dialysis treatment [16]. Globally, there are three main types of dialysate delivery systems for hemodialysis: single-patient dialysis fluid delivery system (SPDDS), central dialysis concentrate supply system (CCDS), and central dialysis fluid delivery system (CDDS). SPDDS are widely used and considered the global standard for dialysis treatment [17]. Using ultrapure dialysate in the CDDS is associated with an improvement in hs-CRP levels compared to standard dialysate, which might confer long-term clinical advantages [18]. CCDS have been implemented in most countries in Europe, the United States, and South Korea, while CDDS are primarily used in Japan [19–22].

In China, centralized dialysate delivery systems have been adopted relatively recently, but their clinical usage has been increasing annually, including both CCDS and CDDS. Currently, CCDS are more widely used. However, CCDS face several problems, including the high cost of new delivery equipment and the need for daily high-temperature disinfection. The resulting significant water and energy consumption and high operating costs are inconsistent with the principles of green dialysis. In contrast, CDDS offer high automation, safety, and efficiency. Their multi-filter design ensures ultrapure dialysate, and their one-touch dead-space-free disinfection is user-friendly [23]. CDDS have been developed over 50 years in Japan, with proven stability, safety, and dialysate purification [24].

A safe, effective, and cost-efficient dialysate delivery system is crucial for China’s healthcare system and ESRD patients. This study aims to evaluate the economic value of CDDS compared to SPDDS in Chinese hemodialysis centers through HB-HTA.

## Methods

This study employed the HB-HTA approach to evaluate the economic value of CDDS versus SPDDS. The economic value was assessed through micro-costing from a hospital perspective, which included cost estimation for CDDS and SPDDS and an economic efficiency analysis under various scales of hemodialysis machine configurations. The analysis encompassed total costs, cost composition, cost savings, and their respective components to identify application scenarios where CDDS demonstrated economic value.

Micro-costing is an accurate method for estimating the costs of medical interventions and is particularly suited for comprehensive economic evaluations [25]. Among the primary methods of medical economic evaluation, micro-costing is especially applicable to studies involving labor-intensive services [26, 27]. Globally, countries and regions such as the United Kingdom, Italy, Spain, Australia, Canada, Hong Kong (China), South Africa, and Malaysia have conducted micro-costing of ESRD-related services. These studies have covered topics such as hemodialysis versus peritoneal dialysis, rural versus urban dialysis settings, nocturnal dialysis, and hospital-based dialysis [28–36]. However, no micro-costing comparing CDDS and SPDDS dialysate delivery systems have been identified.

### Study subjects

This study targeted the nephrology departments of hospitals. Field surveys and questionnaire-based interviews were conducted with management personnel and healthcare professionals from multiple medical institutions in Shanghai. The study clarified the cost components involved in using CDDS and SPDDS for hemodialysis, collected cost data, and calculated the total cost inputs.

### Cost composition

The consideration of costs varies depending on the analytical perspective [37]. Focusing on healthcare institutions in China, we examined direct and indirect costs through a bottom-up approach for detailed cost analysis [38, 39]. The dialysis workflow was outlined based on the *Standard Operating Procedures for Blood Purification (2021 Edition)* and the *Management Standards for Hemodialysis Rooms in Medical Institutions (2019 Revised Edition)*.

Field research was conducted in hospitals in Shanghai with extensive experience in operating CDDS, involving interviews with department directors, medical staff, and engineers. The cost inputs for CDDS and SPDDS were categorized into the following components: labor costs (e.g., equipment operation, consumable handling, waste liquid disposal, and disinfection), equipment costs (e.g., depreciation and maintenance costs), consumable costs (e.g., tubing, heparin, AB solution/powder, saline, dialyzers, ultrapure dialysate, endotoxin filters, disinfectants), and other costs (e.g., storage space for equipment and consumables, training, and costs associated with operational errors). Details are provided in Table 1.

**Table 1:** Cost structure of CDDS and SPDDS

Cost categories	Cost components
Labor costs	Equipment training, preparation of dialysis materials, self-checks until priming, blood drawing, intra-dialysis management, blood return, waste liquid disposal, disinfection, equipment maintenance, troubleshooting, disinfectant replenishment, dialysate handling, etc.
Equipment costs	Equipment purchase price, depreciation period, depreciation cost, failure rate, maintenance costs
Consumable costs	Tubing, heparin, AB powder/solution, saline, dialyzers, waste liquid bags, ultrapure dialysate, peracetic acid, sodium hypochlorite, citric acid, dialysate filters
Other costs	Storage costs for equipment, AB solution/powder, and disinfectants; costs from operational errors

### Data collection

The micro-costing utilized multiple data collection methods [40, 41]. Three hospitals in Shanghai with mature CDDS implementation for maintenance hemodialysis in ESRD patients were selected as sample sites: Baoshan Branch of Huashan Hospital Affiliated with Fudan University, Baoshan Branch of Renji Hospital Affiliated with Shanghai Jiao Tong University School of Medicine, and Jiading District Central Hospital. We mainly relied on administrative databases from these hospitals.

Surveys and interviews included expert consultations and group discussions with nephrology department directors, head nurses, and engineers. The cost input investigation questionnaire was finalized through expert interviews and discussions. The questionnaire was divided into three categories: (1) Department Director Questionnaire: Addressed staff allocation, bed numbers, service volume, etc. (2) Engineer Questionnaire: Covered equipment costs, depreciation, consumable costs, and other expenses. (3) Nurse Questionnaire: Focused on basic labor costs, consumable costs, and other expenses. The questionnaire was designed and distributed via the online tool Wenjuanxing for quantitative data collection. Details were in Supplementary material-Cost Input Survey Questionnaire.

Data analysis was conducted using Excel. A total of 45 questionnaires were collected: 3 from department directors, 3 from engineers, and 39 from nurses (3 head nurses, 36 staff nurses).

Three researchers conducted direct observations in sample hospitals to verify the cost composition and input for CDDS and SPDDS, including staff time allocation.

### Model input parameters

Direct costs included labor costs, equipment costs, and consumable costs. In terms of labor costs, CDDS reduced time input by 11, 27, and 21 min for nurses, engineers, and workers, respectively. In terms of equipment costs, the average costs of single-pump and double-pump hemodialysis machines for SPDDS were considered. Depreciation costs were calculated using the straight-line method, assuming an average depreciation period of 7 years [42]. In terms of maintenance costs, two scenarios were considered: average data from the three sample hospitals (where equipment usage periods varied), and data from one hospital (with approximately 6 years of equipment usage). In terms of consumable costs, average procurement data from the three hospitals were used and cross-referenced with tender prices in the Yaozh Device Database to determine costs. Due to the sensitivity of hospital data, proportional relationships were used instead of raw values. Details are shown in Supplementary material Table 1.

Other cost inputs included training, operational errors, storage space, personnel wages, working hours, and healthcare land costs. Compared to SPDDS, CDDS reduced equipment training time by 4 days, monthly operational errors by 0.11 incidents, and storage space requirements by 8 square meters. In terms of personnel wages, nurses had the highest wages, up to 240,000 RMB/year, while workers had the lowest, approximately 50,000 RMB/year. Working hours were set at 52 weeks/year, 6 days/week, and 8 h/day. Details are shown in Supplementary material Table 2.

### Statistical analysis methods

A micro-cost calculation model was developed using Microsoft Excel 16.80 for Mac. Three scenarios were analyzed:

Scenario 1. Baseline Analysis: the number of hemodialysis shifts per day was set to 2, the cost of hemodialysis machines was set to the average cost of single-pump and double-pump machines, and the equipment maintenance cost data was based on the average maintenance cost from three hospitals.

Scenario 2: Compared to the baseline, the number of hemodialysis shifts per day was increased to 3, while all other parameters remained unchanged.

Scenario 3: Due to differences in equipment usage durations across the three hospitals, scenario analysis was conducted for maintenance cost data. Compared to the baseline, the maintenance cost data was set based on the cost data of one sample hospital, where the equipment had the longest usage period (approximately 6 years).

### Deterministic sensitivity analysis

To understand the sensitivity of model outputs in response to the changes in the values of model parameters, a series of structured and comprehensive one-way sensitivity analysis(OWSA) were adopted to test the robustness of the model outputs. To visualize the changes in output, the model output was measured on the basis of cost savings rate in OWSA. 46 parameters related to cost inputs were included in the OWSA. The variance of all the parameters was set to s varied between − 20% and 20%.

## Results

### Percentage of cost reduction from CDDS

**Fig. 1:**
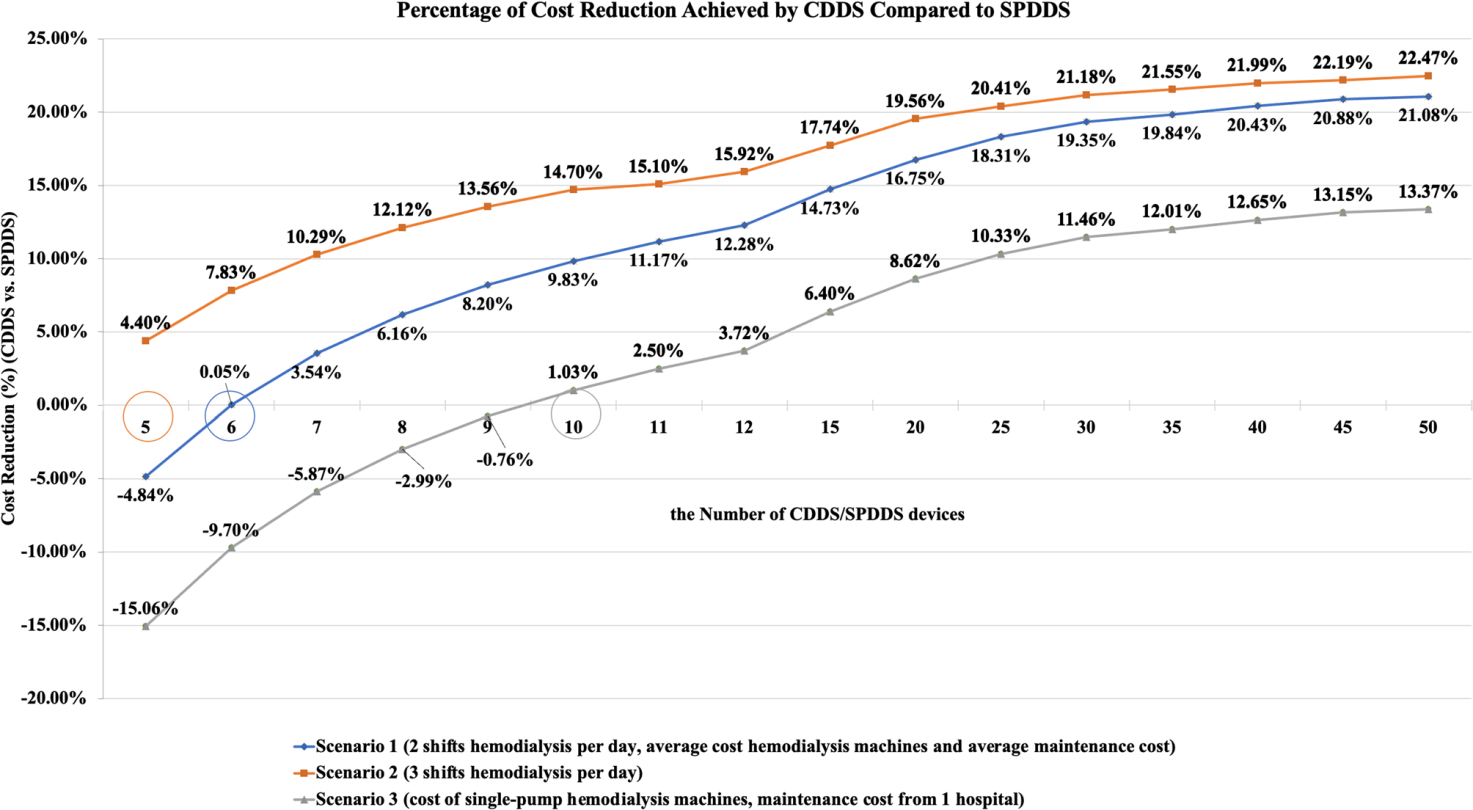
Percentage of Cost reduction achieved by CDDS Compared to SPDDS across various scenarios and hemodialysis service volumes

CDDS had economies of scale. Scenario 1 results show that when dialysis centers equipped both CDDS and SPDDS groups with 6 or more hemodialysis machines, CDDS could reduce costs. As the number of hemodialysis machines increased from 6 to 50, the cost savings with CDDS became more significant. When the number of hemodialysis machines ranged from 6 to 50, the cost reduction rate per treatment session ranged from approximately 0% to 21%. Similar trends were observed in Scenario 2 and Scenario 3. In these scenarios, when equipped with 5 or 10 or more hemodialysis machines, CDDS reduced costs, with cost reduction rates ranging from 4% to 22% and 1% to 13%, respectively. Details are shown in Fig. 1.

**Fig. 2:**
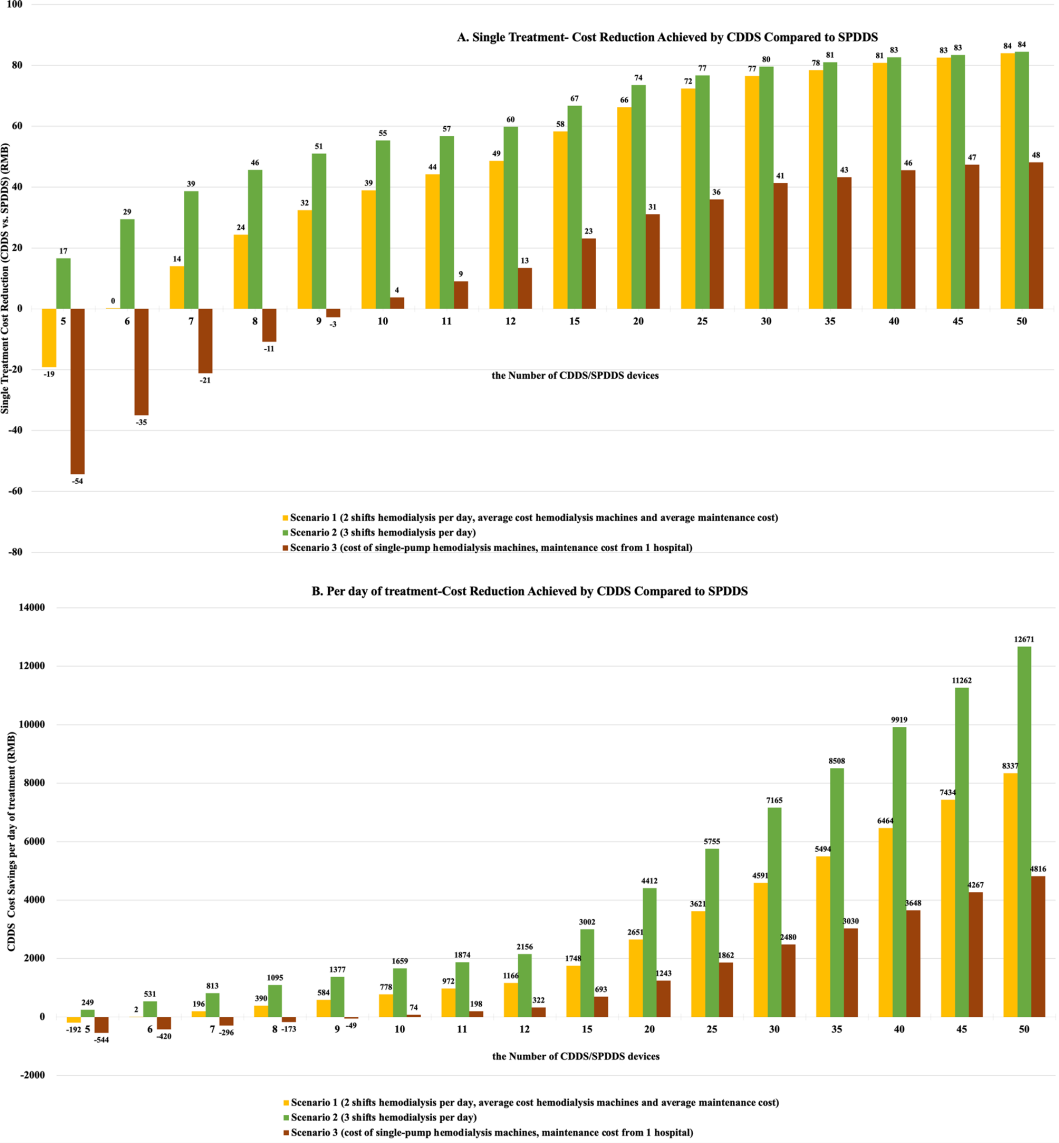

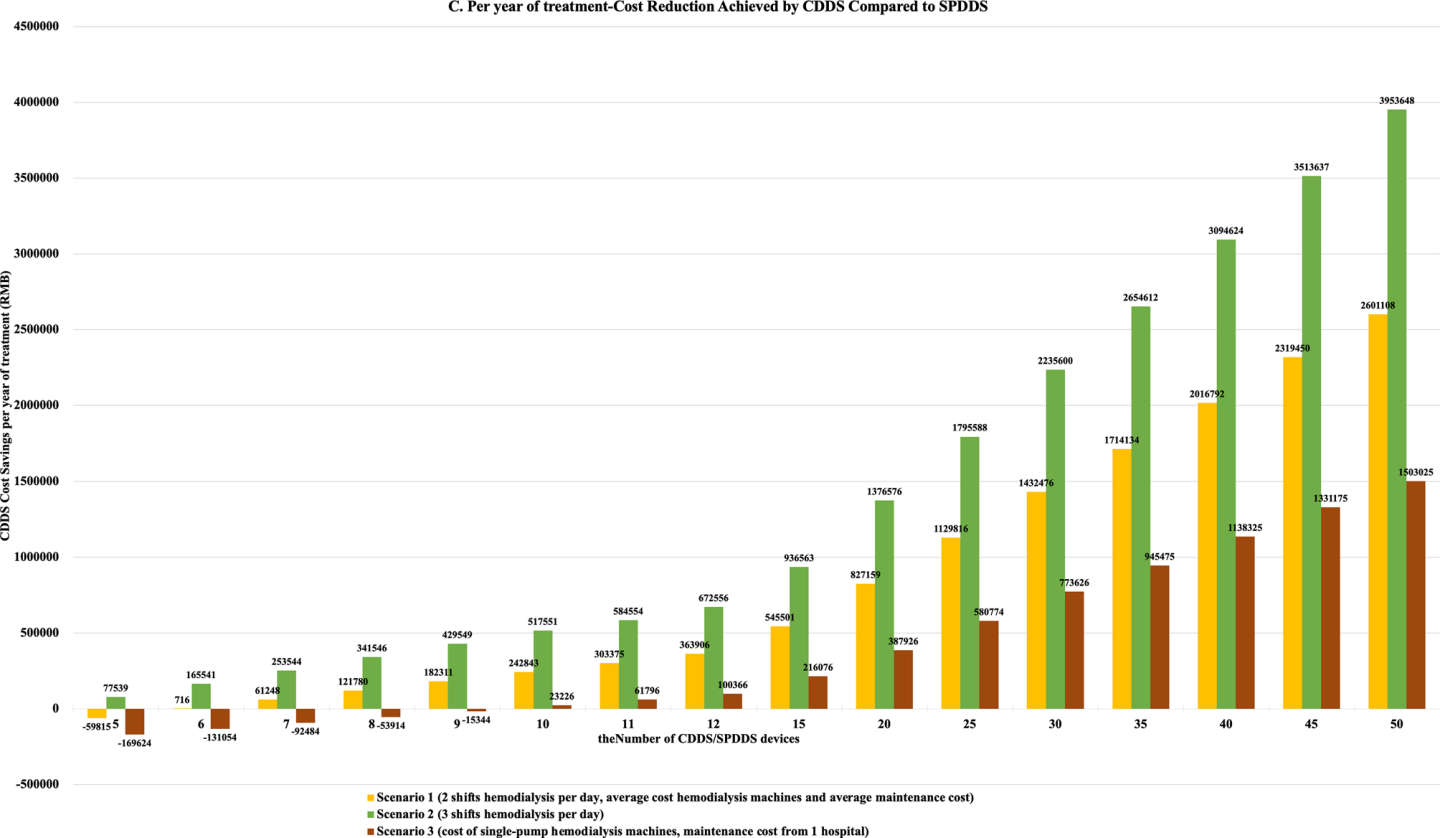
Cost Reduction Achieved by CDDS Compared to SPDDS across various scenarios and hemodialysis service volumes

The results of Scenario 1 show that when dialysis centers equipped both CDDS and SPDDS groups with 6 to 50 hemodialysis machines, the cost savings per treatment session for CDDS ranged from 0 to 84 RMB. The daily cost savings for CDDS ranged from 2 to 8,337 RMB, and the annual cost savings ranged from 716-2,601,108 RMB. Similar results were observed in Scenario 2 and Scenario 3. When equipped with 5 to 50 or 10 to 50 hemodialysis machines, CDDS saved 17–84 RMB (Scenario 2) or 4–48 RMB (Scenario 3) per treatment session, 249 − 12,671 RMB (Scenario 2) or 74 − 4,816 RMB (Scenario 3) per day, and 77,539–3,953,648 RMB (Scenario 2) or 23,226-1,503,025 RMB (Scenario 3) annually. Details are shown in Fig. 2.

### Cost comparison between CDDS and SPDDS

Taking a dialysis center equipped with 50 hemodialysis machines for example, the results of Scenario 1 show that the cost per treatment with CDDS was approximately 312 RMB, while the cost per treatment with SPDDS was approximately 396 RMB. Similar results were observed in Scenario 2 and Scenario 3. The daily cost difference for CDDS ranged from 4816 to 12,671 RMB. The detailed cost differences between CDDS and SPDDS are shown in Table 2.

**Table 2:** Cost comparison between CDDS and SPDDS for a 50-Haemodialysis machine center (RMB)

	Cost Single Treatment(RMB)			Cost per Day of Treatment(RMB)			Cost per Year of Treatment(RMB)		
**Scenario 1**	**CDDS**	**SPDDS**	**CDDS savings**	**CDDS**	**SPDDS**	**CDDS savings**	**CDDS**	**SPDDS**	**CDDS savings**
Labor Cost	18	38	20	1805	3781	1976	563,125	1,179,771	616,646
Equipment Cost	58	59	1	5818	5864	46	1,815,300	1,829,500	14,200
Consumables Cost	235	297	62	23,520	29,695	6175	7,338,317	9,264,789	1,926,472
Traing Cost	1	2	1	67	200	133	21,058	62,462	41,404
Misoperation Cost	0	0	0	1	2	1	166	666	500
Storage Space Cost	0	0	0	2	8	6	472	2358	1886
**Total**	**312**	**396**	**84**	**31,213**	**39,550**	**8337**	**9,738,438**	**12,339,546**	**2,601,108**
**Scenario 2**	**CDDS**	**SPDDS**	**CDDS savings**	**CDDS**	**SPDDS**	**CDDS savings**	**CDDS**	**SPDDS**	**CDDS savings**
Labor Cost	18	38	20	2704	5672	2968	843,542	1,769,656	926,114
Equipment Cost	39	39	0	5818	5864	46	1,815,300	1,829,500	14,200
Consumables Cost	234	297	63	35,092	44,542	9450	10,948,592	13,897,183	2,948,591
Traing Cost	1	2	1	101	300	199	31,587	93,692	62,105
Misoperation Cost	0	0	0	1	3	2	248	1000	752
Storage Space Cost	0	0	0	2	8	6	472	2358	1886
**Total**	**292**	**376**	**84**	**43,718**	**56,389**	**12,671**	**13,639,741**	**17,593,389**	**3,953,648**
Scenario 3	**CDDS**	**SPDDS**	**CDDS savings**	**CDDS**	**SPDDS**	**CDDS savings**	**CDDS**	**SPDDS**	**CDDS savings**
Labor Cost	18	38	20	1805	3781	1976	563,125	1,179,771	616,646
Equipment Cost	58	44	-14	5832	4449	-1383	1,819,450	1,388,167	-431,283
Consumables Cost	235	276	41	23,520	27,603	4083	7,338,317	8,612,189	1,273,872
Traing Cost	1	2	1	67	200	133	21,058	62,462	41,404
Misoperation Cost	0	0	0	1	2	1	166	666	500
Storage Space Cost	0	0	0	2	8	6	472	2358	1886
**Total**	**312**	**360**	**48**	**31,227**	**36,043**	**4816**	**9,742,588**	**11,245,613**	**1,503,025**

Based on 2024 nationwide survey data encompassing 5,164 hemodialysis centers in China, analysis adopted equipment configurations of 23–28 units to reflect the national median (23 units) and mean (28 units) dialysis machine count. The maximum capacity setting of 50 units was implemented to align with the operational realities of tertiary hospitals in Shanghai. Taking a dialysis center equipped with 25 hemodialysis machines for example, the results of Scenario 1 show that the cost per treatment with CDDS was approximately 324 RMB, while the cost per treatment with SPDDS was approximately 396 RMB. Similar results were observed in Scenario 2 and Scenario 3. Compared to 25 hemodialysis machines, the daily cost savings for CDDS ranged from 1862 to 5755 RMB. The detailed cost differences between CDDS and SPDDS are shown in Table 3.

**Table 3:** Cost comparison between CDDS and SPDDS for a 25-Haemodialysis machine center (RMB)

	Cost Single Treatment(RMB)			Cost per Day of Treatment(RMB)			Cost per Year of Treatment(RMB)		
**Scenario 1**	**CDDS**	**SPDDS**	**CDDS savings**	**CDDS**	**SPDDS**	**CDDS savings**	**CDDS**	**SPDDS**	**CDDS savings**
Labor Cost	18	38	20	906	1891	985	282,708	589,885	307,177
Equipment Cost	67	59	-8	3334	2932	-402	1,040,300	914,750	-125,550
Consumables Cost	238	297	59	11,882	14,847	2965	3,707,043	4,632,394	925,351
Traing Cost	1	2	1	34	100	66	10,529	31,231	20,702
Misoperation Cost	0	0	0	0	1	1	83	333	250
Storage Space Cost	0	0	0	2	8	6	472	2358	1886
**Total**	**324**	**396**	**72**	**16,158**	**19,779**	**3621**	**5,041,135**	**6,170,951**	**1,129,816**
**Scenario 2**	**CDDS**	**SPDDS**	**CDDS savings**	**CDDS**	**SPDDS**	**CDDS savings**	**CDDS**	**SPDDS**	**CDDS savings**
Labor Cost	18	38	20	1356	2836	1480	422,917	884,828	461,911
Equipment Cost	44	39	-5	3334	2932	-402	1,040,300	914,750	-125,550
Consumables Cost	236	297	61	17,701	22,271	4570	5,522,680	6,948,592	1,425,912
Traing Cost	1	2	1	51	150	99	15,793	46,846	31,053
Misoperation Cost	0	0	0	0	2	2	124	500	376
Storage Space Cost	0	0	0	2	8	6	472	2358	1886
**Total**	**299**	**376**	**77**	**22,444**	**28,199**	**5755**	**7,002,286**	**8,797,874**	**1,795,588**
**Scenario 3**	**CDDS**	**SPDDS**	**CDDS savings**	**CDDS**	**SPDDS**	**CDDS savings**	**CDDS**	**SPDDS**	**CDDS savings**
Labor Cost	18	38	20	906	1891	985	282,708	589,885	307,177
Equipment Cost	67	44	-23	3341	2225	-1116	1,042,375	694,083	-348,292
Consumables Cost	238	276	38	11,882	13,802	1920	3,707,043	4,306,094	599,051
Traing Cost	1	2	1	34	100	66	10,529	31,231	20,702
Misoperation Cost	0	0	0	0	1	1	83	333	250
Storage Space Cost	0	0	0	2	8	6	472	2358	1886
**Total**	**324**	**360**	**36**	**16,165**	**18,027**	**1862**	**5,043,210**	**5,623,984**	**580,774**

### Cost composition of CDDS and SPDDS

**Fig. 3:**
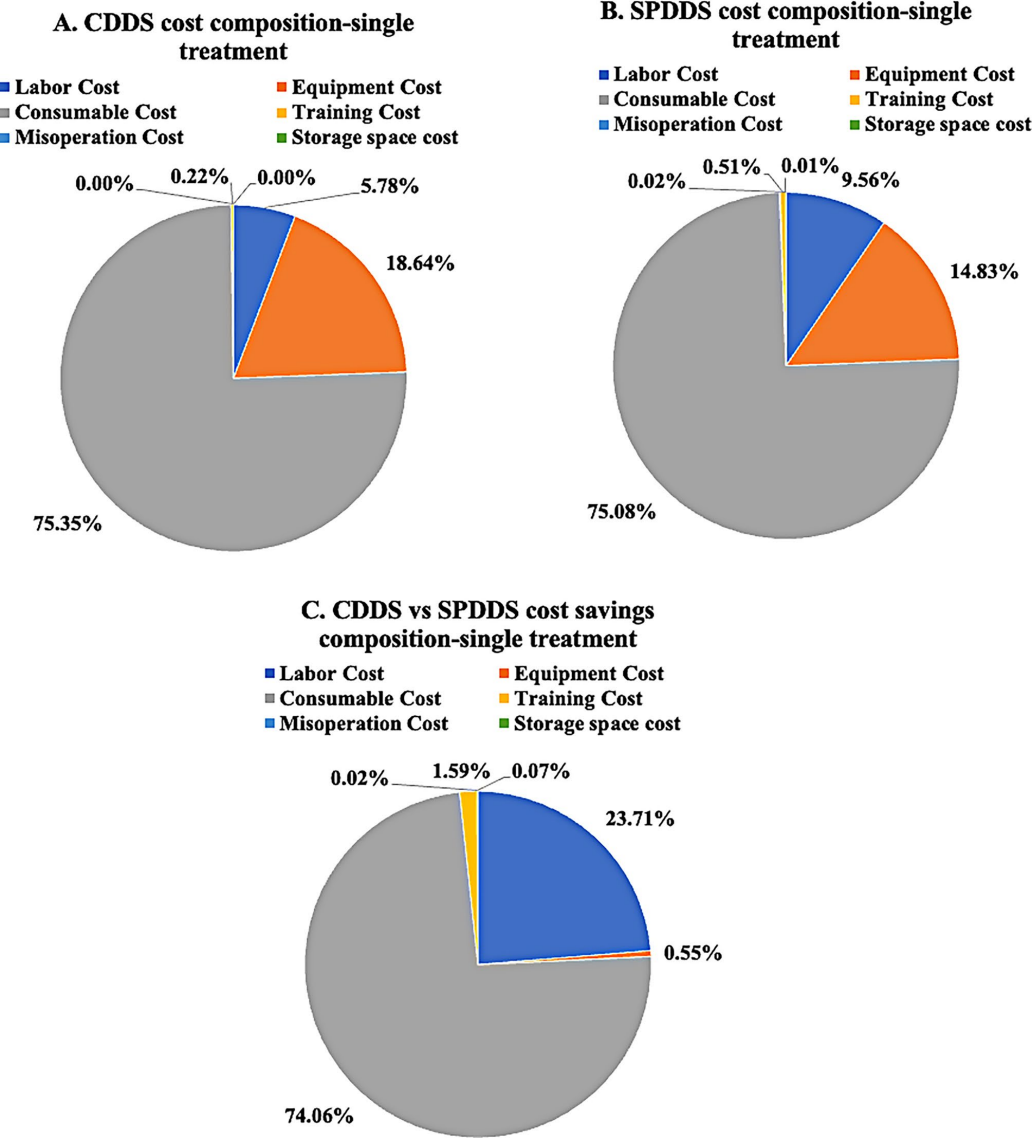
Cost composition of CDDS and SPDDS

Consumable costs accounted for more than 70% of the costs for both CDDS and SPDDS. After analyzing the cost composition for CDDS and SPDDS equipped with 50 hemodialysis machines, it was learned that consumable costs accounted for over 70%, followed by equipment costs at over 15%, and labor costs at 5–10%. Consumable costs accounted for 74% of CDDS cost savings. For a dialysis center equipped with 50 hemodialysis machines, cost composition analysis shows that consumable costs accounted for 74% of the total cost savings, while labor costs accounted for 24%. This indicates that, compared to SPDDS, CDDS significantly reduced the costs of tubing, heparin, AB powder/solution, saline, dialyzers, waste liquid bags, ultrapure dialysate, peracetic acid disinfectant, sodium hypochlorite disinfectant, citric acid disinfectant, and dialysate filters. Moreover, CDDS significantly reduced costs related to nurses, engineers, and workers. Details are shown in Fig. 3.

### Deterministic sensitivity analysiss

**Fig. 4:**
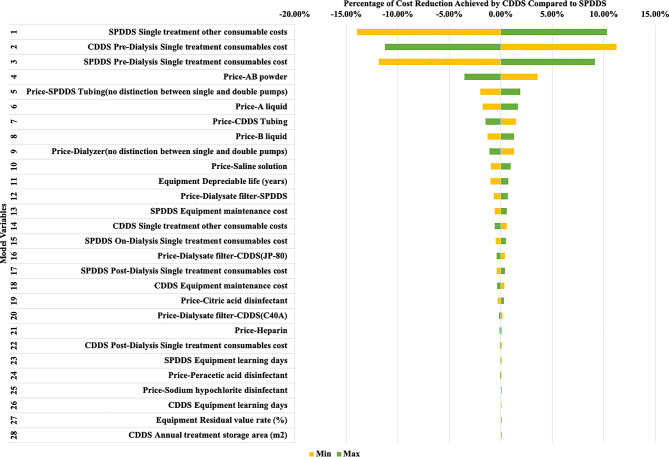
One-way sensitivity analysis tornado diagram - percentage of cost reduction achieved by CDDS compared to SPDDS

Based on the settings in Scenario 1, one-way sensitivity analysis for the percentage of cost reduction achieved by CDDS when equipped with 50 dialysis machines reveals that the significant influencing factors include: consumables costs for CDDS and SPDDS, equipment depreciation period, maintenance costs for CDDS equipment, and equipment learning costs for SPDDS, etc. Regardless of how parameters change, CDDS consistently achieves cost savings, demonstrating the robustness of the cost calculation model in this study. Details are shown in Fig. 4; Table 4.

**Table 4:** Percentage of cost reduction achieved by CDDS compared to SPDDS One-Way sensitivity analysis

No.	Parameters	Min -cost savings(%)	Max -cost savings(%)
1	SPDDS Single treatment other consumable costs	7.13	31.38
2	CDDS Pre-Dialysis Single treatment consumables cost	32.31	9.85
3	SPDDS Pre-Dialysis Single treatment consumables cost	9.24	30.18
4	Price-AB powder	24.64	17.52
5	Price-SPDDS Tubing(no distinction between single and double pumps)	19.09	22.98
6	Price-A liquid	19.34	22.75
7	Price-CDDS Tubing	22.56	19.60
8	Price-B liquid	19.78	22.34
9	Price-Dialyzer(no distinction between single and double pumps)	22.34	19.95
10	Price-Saline solution	20.09	22.05
11	Equipment Depreciable life (years)	20.07	21.78
12	Price-Dialysate filter-SPDDS	20.39	21.75
13	SPDDS Equipment maintenance cost	20.49	21.66
14	CDDS Single treatment other consumable costs	21.65	20.50
15	SPDDS On-Dialysis Single treatment consumables cost	20.59	21.57
16	Price-Dialysate filter-CDDS(JP-80)	21.48	20.67
17	SPDDS Post-Dialysis Single treatment consumables cost	20.69	21.47
18	CDDS Equipment maintenance cost	21.44	20.72
19	Price-Citric acid disinfectant	20.77	21.39
20	Price-Dialysate filter-CDDS(C40A)	21.25	20.91
21	Price-Heparin	21.19	20.97
22	CDDS Post-Dialysis Single treatment consumables cost	21.17	20.99
23	SPDDS Equipment learning days	21.00	21.16
24	Price-Peracetic acid disinfectant	21.14	21.02
25	Price-Sodium hypochlorite disinfectant	21.03	21.13
26	CDDS Equipment learning days	21.11	21.05
27	Equipment Residual value rate (%)	21.06	21.10
28	CDDS Annual treatment storage area (m^2^)	21.08	21.08

## Discussion

CDDS demonstrates significant benefits in ameliorating the patient’s microinflammatory state, as evidenced by marked reductions in both CRP and serum endotoxin levels. It consistently maintains a safe and stable endotoxin level of 0.001 EU/ml. Furthermore, CDDS shows advantages in improving renal anemia, including a lower Erythropoietin Resistance Index (ERI) and reduced requirement for Erythropoiesis-Stimulating Agents (ESAs). These improvements are likely attributable to the positive physiological effects resulting from attenuated microinflammation [17, 18, 43, 44]. Based on HB-HTA, we confirmed the potential advantages of CDDS over SPDDS in clinical applications through a micro-cost analysis. In terms of economic value, micro-cost analysis revealed that CDDS holds economic value in certain specific scenarios. Compared to SPDDS, CDDS can save costs for a nephrology department equipped with 6 hemodialysis machines operating 2 shifts daily and serving 12 patients per day. CDDS brings greater economic benefits to dialysis centers with a larger scale of hemodialysis machines. For a dialysis center with 50 hemodialysis machines, CDDS can save approximately 2,601,108 RMB per year, primarily from savings in consumable usage and labor costs, aligning with the concept of green dialysis. Among sustainable initiatives in haemodialysis, online priming, use of central acid delivery, dialysate autoflow facility, and incremental and decremental haemodialysis showed the most significant savings. CDDS offers potential health benefits and cost savings, with clinical engineers playing a critical role [45–51]. The daily operation of a dialysis center requires close collaboration with clinical engineers. Employing dedicated hemodialysis clinical engineers who conduct regular maintenance and monitoring of dialysis equipment can effectively reduce equipment failures and ensure safe and orderly dialysis treatment.

This study has some limitations. It did not include certain cost inputs that were the same for both the CDDS and SPDDS groups, such as the cost of measuring endotoxins, which means the cost measurement did not account for all costs. Besides, due to data availability restrictions, the study did not include the differences in hospital water and electricity costs, which may result in underestimating the cost and resource savings benefits brought by CDDS [52, 53]. The data parameters in this study were sourced from Shanghai, one of the most economically developed regions in China, so the results are only applicable to cities and regions with similar economic development levels. If the experience is to be further promoted, it is recommended that the micro-cost analysis model developed in this study be updated with new parameters to obtain results that align with the actual conditions of different regions.

## Conclusion

Compared to SPDDS, CDDS demonstrated economic value by reducing consumable and labor costs, making it cost-effective for dialysis centers with 6 or more hemodialysis machines serving at least 12 patients per day. The main sources of cost savings were reductions in consumable and labor costs, with clinical engineers playing an important role in its implementation. In China, CDDS is suitable for promotion in dialysis centers of a certain scale.

## Supplementary Information

Below is the link to the electronic supplementary material.

Supplementary Material 1

Supplementary Material 2

Supplementary Material 3

## Data Availability

The data supporting the findings of this study are available from the corresponding author upon reasonable request.
