# Supplementary material for: Hospital-based health technology assessment of central dialysis fluid delivery system for hemodialysis patients
Source: BMC Nephrol. 2025 Oct 27;26:590. doi: 10.1186/s12882-025-04484-7 (PMC12560572; doi:10.1186/s12882-025-04484-7)
Supplement: Supplementary file 1 — Supplementary Material 1 [file 12882_2025_4484_MOESM1_ESM.doc]

Supplementary material

Table 1. Direct cost input parameters for CDDS and SPDDS

| Cost categories | Cost parameters | CDDS | SPDDS |
| --- | --- | --- | --- |
| Labor costs | Nurse labor time (minutes) | 9.53 | 21.18 |
|  | Engineer labor time (minutes) | 50.3 | 77.7 |
|  | Worker labor time (minutes) | 0 | 21 |
| Equipment costs | Powder dissolution device and dialysate supply system (RMB) | 1,895,000^A^ | / |
|  | Hemodialysis device (RMB) | 190,000^B^ | 196,500^C^/150,000^D^ |
|  | Depreciation period (years) | 7 | 7 |
|  | Residual value rate (%) | 2 | 2 |
|  | Annual maintenance cost per device (RMB) | 1^E^/1.02^F^ | 2.06^E^/1.54^F^ |
| Consumable costs | Tubing (RMB) | 1.00^G^ | 1.66^H^/0.95^I^ |
|  | Heparin (RMB) | 0.35 | 0.35 |
|  | AB powder (RMB) | 1.20 | / |
|  | Solution A (RMB) | / | 1.45 |
|  | Solution B (RMB) | / | 1.09 |
|  | Saline solution (RMB) | / | 0.42 |
|  | Dialyzer (RMB) | 3.82 | 3.82 |
|  | Peracetic acid (RMB) | 2.18 | / |
|  | Sodium hypochlorite (RMB) | 0.43 | 0.43 |
|  | Citric acid (RMB) | / | 2.03 |
|  | Dialysate filter (RMB) | 85.24^J^/715.99^K^ | 45.23 |

Note:

A. The price of the CDDS powder dissolution and dialysate supply system is based on the BC-02 price.

B. The average price of CDDS hemodialysis machines (single-pump and double-pump machines).

C. The average price of SPDDS hemodialysis machines (single-pump and double-pump machines).

D. The average price of SPDDS single-pump hemodialysis machines.

E. The average annual maintenance costs derived from data from three hospitals (sensitive data, original values omitted; proportional relationships are used, assuming the annual maintenance cost per CDDS unit is set at 1, and the annual maintenance cost per SPDDS unit is set at 2.06).

F. Annual maintenance cost data from one hospital (the annual maintenance cost per CDDS unit is set at 1.02, and the annual maintenance cost per SPDDS unit is set at 1.54).

G. The average cost of CDDS tubing is used as the baseline value of 1 (sensitive data; original values are omitted, and proportional relationships are used, with the average cost of CDDS tubing set at 1, and the costs of other consumables compared proportionally to the baseline).

H. The average cost of SPDDS tubing (single-pump and double-pump machines) is 1.66.

I. The average cost of SPDDS tubing (single-pump machines) is 0.95.

J. The average cost of the CDDS dialysate filter ETRF JP-80 is 85.24.

K. The average cost of the CDDS dialysate filter C40A is 715.99.

Table 2 Additional cost input parameters for CDDS and SPDDS

| Cost categories | Cost parameters | CDDS | SPDDS |
| --- | --- | --- | --- |
| Training cost | Training duration (days) | 4.38 | 8.12 |
| Cost of operational errors | Number of operational errors per month (occurrences) | 0.65 | 0.76 |
|  | Treatment delay time per operational error (minutes) | 2.12 | 4.56 |
| Storage space cost | Warehouse area (square meters) | 2 | 10 |
| Personnel salaries | Nurse (RMB/year) | 240,000.00A | |
|  | Engineer (RMB/year) | 220,000.00A | |
|  | Porter (RMB/year) | 53,418.50B | |
| Working hours | Working weeks per year (weeks) | 52 | 52 |
|  | Working days per week (days) | 6 | 6 |
|  | Working hours per day (hours) | 8 | 8 |
| Healthcare land costD | Public administration and public service land, Category 1, Grade 1 (RMB/square meter) | 11,790 | |
|  | Usage period (years) | 50 | |

Note:

A. Nurse and engineer salary data are sourced from hospital surveys and assumptions (sensitive data, based on salary assumptions derived from hospital surveys).

B. Worker salary data are sourced from the *2022 China Statistical Yearbook: 2021 Average Wages of Employed Staff in Urban Non-Private Units* and adjusted based on hospital surveys.

C. Working hours data are sourced from hospital surveys.

D. Healthcare land cost data are sourced from the *Shanghai Benchmark Land Prices for Urban and Rural Construction*.
