# Supplementary material for: Hospital-based health technology assessment of central dialysis fluid delivery system for hemodialysis patients
Source: BMC Nephrol. 2025 Oct 27;26:590. doi: 10.1186/s12882-025-04484-7 (PMC12560572; doi:10.1186/s12882-025-04484-7)
Supplement: Supplementary file 2 — Supplementary Material 2 [file 12882_2025_4484_MOESM2_ESM.doc]

Supplementary material-Cost Input Survey Questionnaire

Cost Input Survey on Central Dialysis Fluid Delivery System for Maintenance Hemodialysis in End-Stage Renal Disease (Nurse Perspective)

Dear Expert,

The Shanghai Health Development Research Center (SHDRC) is conducting a research project titled "Health Technology Assessment of Central Dialysis Fluid Delivery System for Maintenance Hemodialysis in End-Stage Renal Disease." This study aims to calculate and compare the standard cost inputs of the Central Dialysis Fluid Delivery System (CDDS) and Single-Patient Dialysis Fluid Delivery System (SPDDS) for maintenance hemodialysis in end-stage renal disease (ESRD) from the perspective of healthcare institutions. The methodology includes field surveys and questionnaire-based data collection from administrators and medical staff across multiple institutions in Shanghai.

Given your profound expertise in this field, we cordially invite you to participate in this survey. Your insights will serve as critical references for our research. All provided information will remain strictly confidential. Thank you for your invaluable support!

Shanghai Health Development Research Center

(Shanghai Institute of Medical Science and Technology Information)

Shanghai Health Technology Assessment Research Center

1. Respondent Information [Matrix Text Question]

| Field | Response |

|---------------------|-------------------|

| Name | ________________ |

| Age | ________________ |

| Hospital Affiliation| ________________ |

| Years of Experience | ________________ |

| Position/Title | ________________ |

| Contact Number | ________________ |

Human Resource Cost

This section evaluates basic human resource inputs for CDDS and SPDDS during pre-dialysis, intra-dialysis, and post-dialysis phases, including medical waste disposal and disinfection.

2. CDDS – Pre-Dialysis Human Inputs [Grid Text Question]

| Personnel Type | Number of Staff | Working Duration (Minutes) |

|---------------------------------|-----------------|----------------------------|

| Daily dialysis material prep | ______________ | ______________ |

| Dialysis machine self-check/pre-flushing | ______________ | ______________ |

3. CDDS – Intra-Dialysis Human Inputs [Grid Text Question]

| Personnel Type | Number of Staff | Working Duration (Minutes) |

|---------------------------------|-----------------|----------------------------|

| Daily bloodline initiation | ______________ | ______________ |

| Intra-dialysis management | ______________ | ______________ |

| Daily blood return | ______________ | ______________ |

4. CDDS – Post-Dialysis Human Inputs [Grid Text Question]

| Personnel Type | Number of Staff | Working Duration (Minutes) |

|---------------------------------|-----------------|----------------------------|

| Daily fluid drainage | ______________ | ______________ |

| Daily waste fluid disposal | ______________ | ______________ |

| Daily disinfection | ______________ | ______________ |

5–7. SPDDS – Pre/Intra/Post-Dialysis Human Inputs

(Repeat above structure for SPDDS)

Material Costs

This section evaluates basic material inputs for CDDS and SPDDS during pre-, intra-, and post-dialysis phases.

8. CDDS – Pre-Dialysis Materials [Grid Text Question]

| Material | Quantity (Per Day) | Unit |

|------------------------|--------------------|------|

| Tubing (CDDS) | ______________ | _____|

| Heparin | ______________ | _____|

| A Powder | ______________ | _____|

| B Powder | ______________ | _____|

| Normal Saline | ______________ | _____|

| Dialyzer | ______________ | _____|

| Waste Fluid Bag | ______________ | _____|

9–15. CDDS & SPDDS – Intra/Post-Dialysis and Additional Materials

(Repeat structure for intra-dialysis, post-dialysis, and annual consumables)

Other Costs

Includes space costs, training, and operational error costs for CDDS and SPDDS.

16. Space Costs [Grid Text Question]

| Category | CDDS (m²) | SPDDS (m²) |

|-------------------------------------------|-----------|------------|

| Equipment storage (hemodialysis devices) | ________ | _________ |

| Supply storage (A/B powder/liquid) | ________ | _________ |

| Supply storage (sodium hypochlorite) | ________ | _________ |

| Supply storage (citric/peracetic acid) | ________ | _________ |

17. Training & Operational Error Costs [Grid Text Question]

| Category | CDDS | SPDDS |

|-----------------------------------|------------|------------|

| Training duration (days) | ________ | _________ |

| Misoperations (times/month) | ________ | _________ |

| Avg. treatment time increase (min/occurrence)| ________ | _________ |

Cost Input Survey on Central Dialysis Fluid Delivery System (CDDS) for Maintenance Hemodialysis in End-Stage Renal Disease (Engineer Perspective)

1. Respondent Information [Matrix Text Question]

| Field | Response |

|---------------------|-------------------|

| Name | ________________ |

| Age | ________________ |

| Hospital Affiliation| ________________ |

| Years of Experience | ________________ |

| Position/Title | ________________ |

| Contact Number | ________________ |

Equipment Costs

This section evaluates equipment and maintenance costs for CDDS and SPDDS.

2. CDDS Equipment Costs [Grid Text Question]

| Equipment Type | Unit Price (10,000 CNY) | Quantity (Units) |

|----------------------------|-------------------------|------------------|

| Powder Dissolution Unit | ______________ | ______________ |

| Dialysis Fluid Supply Unit | ______________ | ______________ |

| Hemodialysis Device | ______________ | ______________ |

3. CDDS Equipment Depreciation [Grid Text Question]

| Equipment Type | Depreciation Period (Years) |

|----------------------------|------------------------------|

| Powder Dissolution Unit | ______________ |

| Dialysis Fluid Supply Unit | ______________ |

| Hemodialysis Device | ______________ |

4. CDDS Maintenance – Scenario 1 (Annual Contract) [Grid Text Question]

| Equipment Type | Annual Fee (CNY) | Contract Period (Years) |

|----------------------------|-------------------|-------------------------|

| Powder Dissolution Unit | ______________ | ______________ |

| Dialysis Fluid Supply Unit | ______________ | ______________ |

| Hemodialysis Device | ______________ | ______________ |

5. CDDS Maintenance – Scenario 2 (Breakdown Repair) [Grid Text Question]

| Equipment Type | Annual Failure Rate (%) | Cost per Repair (CNY/occurrence) or Primary Failure Types |

|----------------------------|--------------------------|----------------------------------------------------------|

| Powder Dissolution Unit | ______________ | ______________ |

| Dialysis Fluid Supply Unit | ______________ | ______________ |

| Hemodialysis Device | ______________ | ______________ |

6–9. SPDDS Equipment Costs, Depreciation, and Maintenance

(Repeat above structure for SPDDS, including brand-specific details for hemodialysis devices 1–5)

Human Resource Costs

This section evaluates basic human inputs for CDDS and SPDDS operation and maintenance.

10. CDDS – Daily Operational Human Inputs [Grid Text Question]

| Personnel Type | Number of Staff | Working Duration (Minutes) |

|---------------------------------|-----------------|----------------------------|

| Daily A/B powder dissolution | ______________ | ______________ |

| Daily equipment maintenance | ______________ | ______________ |

| Daily troubleshooting/repairs | ______________ | ______________ |

| Daily disinfectant replenishment| ______________ | ______________ |

11. SPDDS – Daily Operational Human Inputs [Grid Text Question]

| Personnel Type | Number of Staff | Working Duration (Minutes) |

|---------------------------------|-----------------|----------------------------|

| Daily A/B liquid transportation | ______________ | ______________ |

| Daily equipment maintenance | ______________ | ______________ |

| Daily troubleshooting/repairs | ______________ | ______________ |

| Daily disinfectant replenishment| ______________ | ______________ |

Material Costs

This section evaluates material inputs for CDDS and SPDDS during pre-, intra-, and post-dialysis phases.

12–19. CDDS & SPDDS Material Costs

(Structure identical to previous nurse version, covering pre/intra/post-dialysis and annual consumables)

Other Costs

Includes space, training, and operational error costs.

20. Space Costs [Grid Text Question]

| Category | CDDS (m²) | SPDDS (m²) |

|-------------------------------------------|-----------|------------|

| Equipment storage (hemodialysis devices) | ________ | _________ |

| Supply storage (A/B powder/liquid) | ________ | _________ |

| Supply storage (sodium hypochlorite) | ________ | _________ |

| Supply storage (citric/peracetic acid) | ________ | _________ |

21. Training & Operational Error Costs [Grid Text Question]

| Category | CDDS | SPDDS |

|-----------------------------------|------------|------------|

| Training duration (days) | ________ | _________ |

| Misoperations (times/month) | ________ | _________ |

| Avg. treatment time increase (min/occurrence)| ________ | _________ |

Cost Input Survey on Central Dialysis Fluid Delivery System (CDDS) for Maintenance Hemodialysis in End-Stage Renal Disease

Questionnaire for Directors of Nephrology Departments

Hospital Name: ________________________

1. Department Staffing

| Category | Number |

|------------------------|--------|

| Physicians | ______ |

| Nurses | ______ |

| Engineers/Technicians | ______ |

| Support Staff | ______ |

2. Department Bed Configuration

| Category | Number |

|------------------------|--------|

| Total Beds | ______ |

| - CDDS-equipped Beds | ______ |

| - Single-Patient Beds | ______ |

3. Department Equipment Configuration

| Category | Number |

|------------------------------|--------|

| CDDS Dialysis Devices | ______ |

| Single-Patient Dialysis Devices | ______ |

4. Department Service Capacity

| Category | Value | Unit |

|----------------------------------|-------|------|

| Annual Working Weeks | ______ | weeks |

| Weekly Working Days | ______ | days |

| Daily Shifts | ______ | shifts |

| Shift Duration | ______ | hours |

| CDDS Patients per Shift | ______ | patients |

| Single-Patient Dialysis Patients per Shift | ______ | patients |
